# Small Molecule-Induced Pancreatic β-Like Cell Development: Mechanistic Approaches and Available Strategies

**DOI:** 10.3390/ijms21072388

**Published:** 2020-03-30

**Authors:** Gitika Thakur, Hyeon-Jeong Lee, Ryoung-Hoon Jeon, Sung-Lim Lee, Gyu-Jin Rho

**Affiliations:** 1 Department of Theriogenology and Biotechnology, College of Veterinary Medicine and Research Institute of Life Science, Gyeongsang National University, Jinju 52828, Korea; gitika18oct@gnu.ac.kr (G.T.); hjlee97@gnu.ac.kr (H.-J.L.); sllee@gnu.ac.kr (S.-L.L.); 2 Department of Cardiovascular Medicine, Mayo Clinic, Rochester, MN 55905, USA; jeon.ryounghoon@mayo.edu

**Keywords:** diabetes, pancreatic differentiation, small molecules, pancreatic beta-like cells, islet organoids

## Abstract

Diabetes is a metabolic disease which affects not only glucose metabolism but also lipid and protein metabolism. It encompasses two major types: type 1 and 2 diabetes. Despite the different etiologies of type 1 and 2 diabetes mellitus (T1DM and T2DM, respectively), the defining features of the two forms are insulin deficiency and resistance, respectively. Stem cell therapy is an efficient method for the treatment of diabetes, which can be achieved by differentiating pancreatic β-like cells. The consistent generation of glucose-responsive insulin releasing cells remains challenging. In this review article, we present basic concepts of pancreatic organogenesis, which intermittently provides a basis for engineering differentiation procedures, mainly based on the use of small molecules. Small molecules are more auspicious than any other growth factors, as they have unique, valuable properties like cell-permeability, as well as a nonimmunogenic nature; furthermore, they offer immense benefits in terms of generating efficient functional beta-like cells. We also summarize advances in the generation of stem cell-derived pancreatic cell lineages, especially endocrine β-like cells or islet organoids. The successful induction of stem cells depends on the quantity and quality of available stem cells and the efficient use of small molecules.

## 1. Introduction

Diabetes mellitus (DM) is a group of metabolic diseases diagnosed by chronic hyperglycemia which is caused by insufficient insulin production or the destruction of pancreatic beta (β)-cells. Diabetic people have high risk of developing a number of serious and potentially lethal health issues resulting in reduced quality of life, higher medical care costs, and increased death rates [1]. Continuous high glucose levels in the blood cause damage to blood vessels, affect the eyes, kidneys, heart, and nerves, and lead to numerous complications [2]. In 2019, it was estimated that 463 million people were living with diabetes, and this number is expected to reach 578 million by 2030 and 700 million by 2045 [3]. Diabetes remains undiagnosed in almost half of those that have it (50.1%). Moreover, global impaired glucose tolerance prevalence was estimated to be 7.5% in 2019, or 373.9 million people, and is projected to reach 8.0% (453.8 million) by 2030 and 8.6% (548.4 million) by 2045 [4]. As T1DM is characterized by an inadequacy of insulin due to autoimmune devastation of pancreatic β-cells [5], the current available strategy to cure the disease is exclusively dependent on the exogenous uptake of insulin. For this, patients need to inject exogenous insulin several times per day throughout the whole lives. To minimize those difficulties and to improve their quality of life in the long term, cell therapy using insulin-secreting β-like cells may be one of the best treatments. Although islet transplantation can be an effective therapy for type 1 diabetes, a severe shortage of donors, immunosuppression, and patient specificity issues have led researchers to seek alternative approaches to overcome life-long exogenous insulin dependency and transplantation complications. The available approaches can neither cure nor completely manage the difficulties related to DM, which leads to losses of life. Moreover, current methods inflict high economic and social–mental stress on the concerned parties.

Transplantation of the pancreas or islet [6] can control normoglycemia [7] and eliminate the need to use exogenous insulin. This procedure results in better glycemic control and can limit patients’ exogenous insulin dependency for longer periods, thereby improving quality of life [8]. Still, there are some shortcomings, like the dearth of cadaveric donors in comparison to the large number of diabetic patients, and immunosuppression issues [9]; therefore, alternative sources of surrogate cells are required.

Stem cells (hiPSCs: human induced pluripotent stem cells, hESCs: human embryonic stem cells, hMSCs: human mesenchymal stem cells) are a very alluring alternate source of surrogate β-like cells due to their ability to differentiate into all major somatic cell lineages [10,11]. As discussed above, cellular therapy is an efficient method to cure diabetes and many other diseases related to the pancreas, which may be achieved by differentiating pancreatic β-like cells through different approaches, such as growth factors, small molecule-induced differentiation, reprogramming, and epigenetic modifications. Many scientists have reported important genes and signals for the pancreatic lineage, and these have been effectively used to form β-like cell lineages in vitro from stem cells [12,13,14]. The approach which reproduces normal pancreatic β-cell development has achieved functional insulin-producing β-like cells from stem cells. Many researchers have acted in accordance with directed differentiation techniques, whereby cells are exposed to numerous signaling molecules and growth factors at particular stages with specific doses in a specific sequence for the successful differentiation of endocrine cells (ECs) [15,16,17]. Nonetheless, many studies have resulted in the production of polyhormonal cells, which lack β-cell transcriptional factor expression and are unable to secrete insulin in response to glucose challenge in vitro [17,18,19,20,21]. Such insufficient functionalities may have been due to an inappropriate surrounding environment of hPSC-derived insulin-secreting cells, unlike native β-cells. The need of longer durations to achieve functional ability may be due to immature ECs. Additionally, researchers also showed that hESC-derived endocrine cell clusters (ECCs) after transplantation secrete insulin, and that glucose homeostasis could be attained within a short period [22]. This means that the formed ECCs were mature, i.e., they did not require extra time to achieve functional insulin secretion ability. Furthermore, an in vivo mouse model with ECCs grafts showed mature β-like cell markers, as well as valuable C-peptide secretion [22]. This indicated that ECCs could control glucose levels quickly and effectively in diabetic patients, but only for a short time. Kim et al. presented the possibility of using hPSC-derived pancreatic β-like cells to cure diabetes [22].

Until now, prodigious accomplishments have occurred in the establishment of differentiation protocols, where signaling molecules and growth factors were used in a time-dependent manner, leading to the generation of β-like cells in vitro which expressed mature β-cell markers and improved hyperglycemia in diabetic models [15,17,19,23,24]. Furthermore, the induced β-like cells showed gene expression patterns and characteristics like insulin generation and secretion, both in in vivo and in vitro conditions, which were similar to native pancreatic β-cells [15,17,19,23]. Some studies have shown that stem cell-derived pancreatic progenitors (PPs) take 3–4 months for maturation after transplantation; this is due to the immaturity of the in vitro differentiated β-like cells [22], and recent advancements in generating β-like cells in vitro have significantly shortened the time required for the cells to become fully functional before transplantation [22,24,25].

Still, there are some drawbacks in the available differentiation strategies; to improve this, approaches such as the development of appropriate microenvironments with oxygen gradients, nutrients, favorable extracellular matrices [26], metabolites, and cheaper, small molecules or growth factors are required for efficient cell therapy. Different strategies, such as improvements of the culture media by adding necessary factors and using bio-compatible scaffolds can be attempted to achieve long-term functional ability of pancreatic β-like cells/islet organoids after transplantation.

In this review article, we mainly focus on small molecules-based strategies to differentiate stem cells into pancreatic β-like cells to treat DM. Small molecules are more advantageous than proteins/growth factors due to their cell-permeable and non-immunogenic properties [27]. Furthermore, they are relatively cost effective, stable, easy to synthesize, and readily standardized. The small molecule controlled activation or inhibition of target proteins can be reversible and adapted through adjustments in concentration [28]. It is important, however, not to underestimate serious issues related to low differentiation efficiency, poor quality, and the maturity of the differentiated cells. A selection of suitable inducers is important for the cellular differentiation of β-like cells.

## 2. Small Molecules

Small molecules are low molecular weight (<900 Daltons) organic compounds which can modulate cell differentiation. They can efficiently promote cell differentiation and govern other cellular activities like common promoters and growth factors which are expensive, and can be harmful. Small molecules are cell permeable chemical compounds which are generally safer, inexpensive, pure, amenable to scaling up, and are devoid of animal derived products which makes them more suitable candidates to be used in in vitro practices [29,30,31,32]. The main motivation behind using small molecules is that they can be synthesized in large amounts with higher purity and stored in a way that ensures their reproducible activity [30]. Small molecules have been efficiently used in differentiation processes, as they regulate cellular processes by inflecting signal transduction pathways, metabolism, or gene expression. They can be integrated with high purity and quantity, as well as smoothly applied or evacuated, making them well-suited to therapeutic applications. They can also support the self-renewal of ES cells [33,34], and are not only useful in achieving the desired cell types for various functions in vitro, but can also be further used as drugs to cure damaged cells to regenerate in vitro [29].

Small molecules are used to attain many different cell lineages such as osteoblasts [35], neurons [36], cardiomyocytes [37], hepatocytes [38], and pancreatic β-like cells [19]. These can serve as a tool to replace current proteins and induce differentiation of stem cells (PSCs and MSCs), and can also effectively act on target proteins, thereby playing a key role in modulating different signaling pathways [39].

The search for functional differentiated cells is compelling researchers to screen and identify suitable small molecules by which to direct specific cell lineages. The use of small molecules has made it easier to recapitulate in vivo environments by the activation/inhibition of cell-specific signaling. During the screening of thousands of compounds, some cell permeable small molecules are identified that direct the differentiation of stem cells into the pancreatic β-like cell lineage. These compounds, which induce a high percentage of stem cells to form definitive endoderms (DEs) and further stages during pancreatic β-like cell differentiation in a stepwise manner, have achieved higher efficiency than other factors which are commonly used as inducers of endoderms. The application of small molecules to differentiate stem cells into endoderms and PPs represents a step toward the production of the desired stem cell derivatives. In 2009, Borowiak et al. screened about 4000 compounds and found only two cell permeable small molecules, i.e., inducers of definitive endoderm1/2 (IDE1/2), that resulted in the efficient differentiation of DE cells, and ultimately, in the production of insulin-producing cells [39].

Stem cells can be isolated and cultured from blastocyst, skin, blood, urine, dental tissue, placenta, umbilical cord, whartons’ jelly, and bone-marrow. Almost all stem cell-derived insulin-producing cell protocols involve the integration of small molecules. Based on the different sources of stem cells (ESCs, iPSCs, and MSCs), the pancreatic developmental procedure after achieving the definitive endodermal stage remains the same. The involvement of small molecules depends upon the choice of researchers to control a particular signaling pathway (Table 1). Additionally, pluripotent cells have teratoma-related ethical issues, while MSCs have no risk of teratoma formation and have unique immune-modulation properties. So, out of PSCs (ESCs, iPSCs) and MSCs, the latter is the best option for the generation of differentiated cells for cellular therapy. This review mainly discusses the use of small molecule-based pancreatic β-like cell differentiation; immune-modulation properties are not discussed here in details.

**Figure 1:**
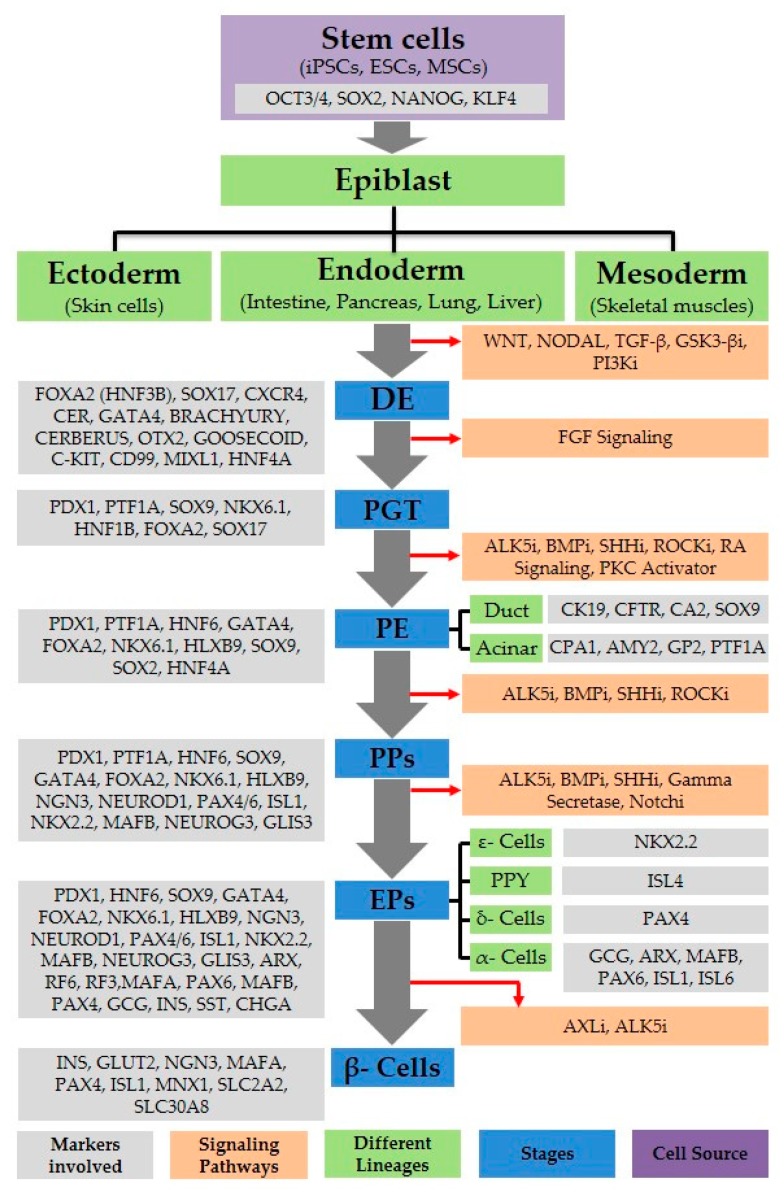
Pancreatic beta-cell development. A flow diagram depicting various lineages of stem cell differentiation toward pancreatic cell progeny. Signaling pathways, as well as markers for various cell types, are shown. i: Inhibitor; DE: Definitive endoderm; (PGT) posterior gut tube, PE: pancreatic endoderm, PPs: pancreatic progenitors; EPs: endocrine progenitor cells.

Many researchers have used various small molecules to induce the differentiation of stem cells (ESCs, iPSCs, and MSCs) into insulin expressing cells at different stages of development, and have analyzed pancreatic endocrine markers SOX17, FOXA2, PDX1, NKX6.1, Insulin, and C-Peptide (Figure 1 and Table 1). Additionally, factors like sodium butyrate, TSA, TMP269, RG, Par, and NECA amplify the definitive endodermal differentiation of stem cells (ESCs, iPSCs, and MSCs) [40,41,42]. Along with their cost effective use and ease of delivery and to removal, they also minimize the risk of contamination. Moreover, small molecules ameliorate serum-free approaches to stem cell differentiation.

## 3. Signaling Pathways

Different signaling pathways, including Transforming Growth Factor beta (TGF-β), WNT, Fibroblast growth factors (FGF), notch, Sonic hedgehog (SHH), Retinoic acid (RA), and Bone morphogenic protein (BMP), and their relevant receptor activation processes, are involved in pancreatic β-like cell differentiation from stem cells (ESCs, iPSCs, and MSCs) (Figure 1). Attention is widely concentrated on the identification of significant signaling pathways and molecules involved in pancreatic β-like cell development. The TGF-β superfamily of Activin performs a crucial function during the starting phase of pancreatic development. As TGF-β is crucial for β-cell function, it governs the differentiation and propagation of cells by autocrine and paracrine signals [47]. Down regulation of TGF-β signaling impedes embryonic, pancreatic β-like cell differentiation and development [13]. TGF-β signaling enhances EC differentiation and maturation, and inhibits acinar cell growth [48]. Activin/Nodal signaling is controlled by suppressing Phosphoinositide 3-kinase (PI3K) signaling pathway (Figure 1) [49]. Recently, β-like cell differentiation through the use of various small molecules has received significant attention, but some signaling pathways involved in different development stages are still unknown. The WNT signaling pathway also plays an important role in pancreatic development; it is mainly responsible for migration, cell polarity, and proliferation, and promotes self-renewal. Hence, it is needed during the initial stage to induce definitive endoderm (DE) differentiation. The formation of the pancreas in humans and mice can be promoted by SHH inhibition [50]. In the early stages of pancreatic development, it is necessary to block BMP signaling, whereas during PP differentiation, its activation is compulsory [40,51]. Notch signaling is involved in maintaining the survival, cell cycle, and differentiation of multicellular organisms. In mammals, severe defects in embryonic development and tissue homeostasis are due to the loss of notch signaling, and its down-regulation is associated to multiple developmental and physiological defects [12,52]. During normal pancreatic development, various aspects of the notch pathway are essential [12,53]. Diverse mutations in the genes (Rbp-Jk, Delta1, and Hes1) of notch signaling result in the elevated expression of NGN3, which leads to the reduction of precursor cells (Pancreatic progenitors) followed by pancreatic hypoplasia [12,52].

## 4. Protocols for the Differentiation of PSCs/iPSCs/MSCs into Functional Pancreatic β-Like Cells in a Stepwise Manner

**Figure 2:**
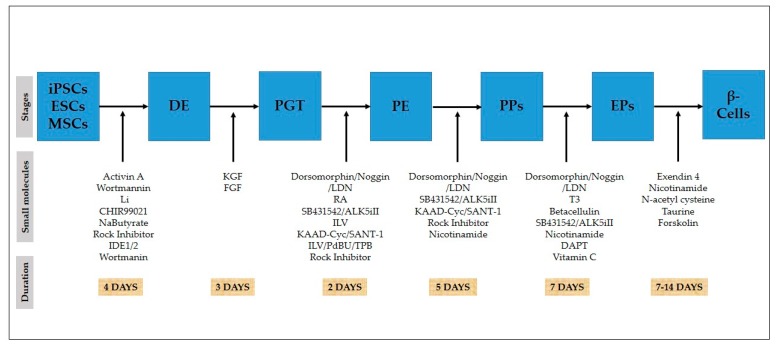
In vitro differentiation of stem cells towards pancreatic β-cells. Timeline of differentiation of stem cells transforming small molecules into β-cells.

The differentiation of stem cells (ESCs, iPSCs, MSCs) into pancreatic β-like cells in a stepwise manner, as shown in Figure 2, has been reported in various studies. It is achieved by mimicking the process of pancreatic development [19,51].

In mammals, during gastrulation, the mesoderm and DE are derived from the epiblast. Then, flat DE pleats form a primitive gut tube in which the foregut, midgut, and hindgut can be recognized in an anterior–posterior orientation, surrounded by the notochord and mesenchyme. The foregut is divided into the anterior and posterior foregut, where the anterior part includes the esophagus, thymus, liver, lungs, pancreas, and thyroid [54,55,56]. Despite the specification programs of ventral and dorsal anlagen, the Activin and FGF2 secreted by notochord suppresses SHH signaling, leading to the appearance of PDX1-positive (PDX1 is strongly expressed in the nucleus) epithelium, which is the main early marker of pancreatic differentiation [14].

For a better understanding of human pancreatic development, it is essential to develop a procedure for differentiation to form efficient β-like cells in vitro. Methods for differentiation should be based on knowledge of early pancreatic development, the successive expression of transcription factors [57], and the signaling pathways [58] involved in β-like cell formation (Table 2). To improve the differentiation of insulin-producing cells, combinations of different key cytokines, small molecules, and growth factors are used (Table 3), which regulate the necessary signaling pathways specific to different stages of pancreatic β-cell development, i.e., the DE, posterior gut tube (PGT), pancreatic endoderm (PE), PPs, endocrine progenitor cells (EPs), and finally, β-like cell lineage differentiation.

### 4.1. Differentiation of Stem Cells into Definitive Endoderm: Activin-A and CHIR99021

DE specification is a prerequisite step for the development of organs, i.e., mainly liver and pancreas. In vitro differentiation steps mimic the developmental stages that take place during the development of pancreatic β-cell lineages in humans and mice. Thus, DE differentiation is based on imitating TGF-β, WNT, and Nodal signaling, and by inhibiting the PI3K and GSK-3β signaling pathways. The generation of DE is followed by PDX1 and insulin expressing cells. Study of developmental biology should be followed.

Activin proteins are members of the TGF-β superfamily which are widely used by various researchers to achieve DE [59,62,65,72,75,76,77]. Moreover, in order to increase the number of DE cells, GSK3-β and PI3K activity is suppressed by different inhibitors, i.e., LY294002, Wortmannin, CHIR99021, MCX-928, and by the enhanced induction of DE marker expression, i.e., FOXA2, SOX17, and CXCR4 [23,49,61].

Treatment with Activin-A in combination with CHIR99021 [17,19,22,23,59,61,78], which is a highly selective inhibitor of GSK3β and GSK3α, induces efficient differentiation of stem cells into DE, resulting in significantly increased expression of FOXA2 and SOX17 (Endodermal markers). CHIR99021 is preferred over WNT3A, due to its high efficiency, stability, and cost, for the differentiation of stem cells into DE [19]. MCX-928 has also been used as GSK3βi to induce DE differentiation [23,30].

Through the treatment of Activin-A, LiCl, and CHIR99021 for 1 day followed by Activin-A alone for 4 days, a relatively high percentage of CXCR4-positive cells (94%) were achieved, as demonstrated by the higher expression levels of SOX17, FOXA2, and GATA4 at the mRNA and protein level [22]. IDE1/2 (Inducers of definitive endoderm 1 and 2) was identified by Meltons’ Lab and Borowiak et al. IDE1/2 was shown to direct the differentiation of humans and mouse stem cells into DE cells (70%–80% endoderm cells than that of Activin-A and Nodal), leading to the activation of a TGF-β pathway, as demonstrated by SMAD2 phosphorylation [38,59,60]. Jiang et al. checked the effect of Activin-A alone and in combination with sodium butyrate. Elevated expression of SOX17, FOXA2, and HNF4A were induced in the latter case [69].

In 2011, Xu et al. stated that the treatment of stem cells with bFGF, Activin-A, and BMP4 (collectively referred to as FAB) leads to strong commencement of DE related genes, including GSC, FOXA2, MIXL1, and SOX17. When embryoid bodies (EBs) made from stem cells are treated with FAB instead of Activin-A alone, the expression of the FOXA2, SOX9, and PDX1 pancreatic lineage markers was observed [76,79].

Nonmetastatic cells 2 protein (NME2) is abundant in undifferentiated PSCs; upon differentiation, its levels reduce, indicating its significant function in the maintenance of undifferentiated cells. Stauprimide cooperates with Activin-A by communicating with the nodal signaling pathway. Treatment with stauprimide alone or with Activin-A leads to DE cells that are double positive for N-Cadherin and SOX17. The low percentage (10%) of N-Cadherin and SOX17 positive cells can be induced when cells are treated with Activin-A alone, whereas the combination of Activin-A and stauprimide significantly elevates the N-Cadherin and SOX17 positive population (60%). In comparison, DMSO- and Stauprimide-treated cells are identical in relation to N-Cadherin and SOX17 expression when Activin-A is not present [70,71].

### 4.2. Posterior Gut Tube: KGF

Once the DE stage is achieved, the next step is to differentiate DE into PGT. In 2019, Yabe et al. treated cells with a combination of sodium pyruvate and insulin, transferrin, selenium, and ethanolamine solution (ITS-X solution) [80]. They showed that a high dose of FGF4 promotes endodermal cell fate, whereas lower doses induce anterior/pancreas-duodenal cell fate [81]. Furthermore, a lower FGF2 concentration leads to hepatic lineage, while moderate and high FGF2 levels induce pancreatic and intestinal progenitors, respectively [66]. The induction of DE cells by the Keratinocyte growth factor (KGF) is more efficient than with FGF10 [76,82]. As a result, transcription factors HNF1β and HNF4α are highly expressed in PGT cells. Additionally, transcription factors FOXA2 and GATA4 are strongly expressed in stage 1, which remains the same throughout KGF induction. Recently, several researchers have used KGF alone or in combination with other small molecules to induce PGT [17,65].

### 4.3. Pancreatic Endoderm: RA, LDN, SB431542, and SANT-1

PE formation has been characterized by the expression of PDX1 and NKX6.1 transcription factors. RA is widely used in differentiation media, which leads to pancreatic endoderm formation [22,61,62,83,84]. Several groups have shown that Noggin, i.e., a BMP signaling inhibitor, suppresses hepatic lineage differentiation; it is used in combination with RA to enhance pancreatic differentiation [18,40,51,65,66,83]. Some researchers have used LDN193189 as a BMP antagonist [25,67]. As discussed above, the inhibition of SHH signaling is mandatory to promote pancreatic lineage development; therefore, various researchers have used different antagonists of SHH, such as cyclopamine and SANT1 [22,23,50,72,85]. The treatment of PE cells with a cocktail of RA, Dorsomorphin, SB432942, bFGF, and KAAD-cyclopamine results in the robust expression of PDX1. The expression of PDX1, HNF1β, and SOX9 is significantly enriched in PE cells compared to DE cells [21]. When PGT is treated with RA together with FGF10 and KAAD-cyclopamine, cells rapidly show higher expressions of HNF6, PDX1, HNF4A, and HNF1β, i.e., the main markers of PE. Interestingly, the absence of RA leads to the reduced expression of NGN3 and insulin in later stages of development. This is constant in several models, revealing the necessity of RA signaling for pancreatic development [55,86]. In addition, if KAAD-cyclopamine is excluded at this stage, the level of insulin expression becomes lower in the later stages due to the effect of SHH signaling, which promotes stomach and duodenal endoderm, as will be explained earlier. This reveals the necessity for low SHH signaling during pancreatic bud specification [87].

PDX1 gene expression is essential for pancreatic development, because mutations of this gene in humans result in the failure of pancreatic development, and ultimately, in diabetes [88,89]. This gene also plays an essential role in the pancreatic development of mice, as its loss leads to pancreatic agenesis [90]. For PDX1 induction, the activin signal from notochord, RA signaling [91], and SHH inhibition are necessary in dorsal endoderm [14,87], followed by endothelial cell signaling, which is crucial for maintaining the expression of PDX1 in the dorsal pancreas [92]. In humans, the notochord and dorsal endoderm are both in contact with each other, which is why there is no SHH in the dorsal human foregut endoderm [93]. After PGT induction, PKC activator, FGF7, the BMP inhibitor LDN, Vitamin C (Vit C), RA, and the SHH inhibitor SANT1 are sustained to produce PDX1-positive PPs in vitro [20,23,25,94]. Indolactam V functions via the calcium-dependent PKC signaling pathway [14,74]. Recently, several researchers have applied a stepwise protocol (Figure 2), using optimized doses of FGF, along with other factors like SB431542, RA, EGF, Noggin, and KAAD-cyclopamine after the formation of foregut, and have achieved increased expression of PDX1-positive cells, indicating that FGF is essential for PDX1 expression [18,22,40,51,65,69,72]. Thus, the protocol partially mimics the methods of pancreatic induction by direct SHH inhibition, rather than imitating signals of the notochord, which seems to neglect uncertain endothelial signals. There are not many selective markers for the dorsal pancreatic bud, but the dorsal endoderm markers HLXB9 and MNX1 need further analysis in human systems [95,96]. The use of Vit C increases the number of cells and prevents EPs specification in the early stages of 2D culture [23].

### 4.4. Pancreatic Progenitors: DAPT and TPB

The next step is the generation of PPs, which is carried out in the absence of RA. Notch signaling governs the subsequent cell fate outcomes which are appropriate for specialized tissue formation, including β-like cell generation, and prevents immature endocrine differentiation. In order to achieve a large number of ECs in vitro and to expand the PDX1-positive population of progenitor cells, EGF treatment could be beneficial [51]. After FGF10 and EGF treatment, notch signaling inhibition seems to be an important factor regarding endocrine cell fate. As for the formation of ECs, NGN3 is an essential transcription factor, which is suppressed by notch signaling. Therefore, notch signaling can be inhibited by gamma-secretase inhibitor and N-[N-(3,5-difluorophenacetyl)-L-alanyl-S-phenylglycine t-butyl ester (DAPT), but this has a negligible impact on the differentiation of ECs [18]; this may be due to nonspecific inhibition. Further differentiation into PPs is achieved by applying mixture of several drugs, i.e., ALK5 (TGF-β receptor) inhibitor II (ALK5iII: Activin receptor-like kinase 5 inhibitor II), TPB (((2S,5S)-(E,E)-8-(5-(4-(trifluoromethyl)phenyl)-2,4-pentadienoylamino) benzolactam), BMP receptor inhibitor (FGF7), and LDN; which lead to elevated expression of NGN3, which is mandatory for β-like cell maturation and differentiation. On the other hand some researchers have used Heparin, Vit C (which prevents polyhormonal cells formation), Betacellulin (an EGF receptor ligand and the growth-promoting factor that maintains NKX6.1 expression), Gamma-secretase inhibitor (which suppresses notch signaling) and Triiodothyronine (no clear evidence exists of its effect on pancreas development) lead to up-regulated NGN3 expression, resulting in a cell population which coexpresses NKX6.1, NEUROD1, PDX1, and NKX2.2 [17,23,94].

NKX6.1 and PDX1 coexpression is significant for PPs, PDX1-positive cells which do not express NKX6.1-positive are duodenal cells [15,65,97]. If PDX1-positive cells express NKX6.1, then a large number of diffferentiated cells leads to the elevated expression of mature β-like cells after transplantation [15]. Recently, it has been shown that NKX6.1 expression at the EP stage will be sufficient to form β-like cells [98]. Glycoprotein 2 which is a cell surface protein is particular to PPs; it is analyzed by the expression of PDX1-positive/NKX6.1-positive progenitor markers [66,99]. It may be a convenient marker for cell sorting, as it is considerably more definite than the CD142 marker [100].

### 4.5. Endocrine Progenitors: Nicotinamide, N-acetyl Cysteine, and Exendin-4

The formation of insulin-producing cells occurs through the directed differentiation of EPs into functional and specialized hormone producing cells. A large number of biologically active compounds have been already used in published differentiation protocols (Figure 2), yet a high percentage of functional β-like cells have not been attained to date. D’Amour et al. used a cocktail of various maturation factors such as Hepatocyte growth factor (HGF), Exendin-4, insulin growth factor 1, and B27 at the time of terminal differentiation, but when these factors were omitted, only minor effects were observed on differentiation [18], which means that some other crucial factors were required. On the other hand, it was concluded that the application of Nicotinamide and Betacellulin to D’Amours’ protocol results into the steady expression of PDX1, and led to the production of insulin [101]. In vitro differentiation protocols can lead to the production of insulin cells, but they are commonly nonfunctional and immature. In conclusion, the terminal differentiation step was removed from the procedure in vitro where PPs were allowed to mature into functional cells after transplantation into streptozotocin-induced hyperglycemic mice in vivo [65].

Finally, ECs have been developed from EP cells, where the expression of insulin, somatostatin, pancreatic polypeptide, and C-peptide was clearly observed. When EP cells were treated with a cocktail of SB431542, Nicotinamide, Ascorbic acid Dibutyryl-cyclic AMP (Db-cAMP), Exendin-4, and Dorsomorphin [22], the coexpression of MAFA and insulin with a low level of NKX6.1 were seen in ECs, which means that differentiated ECs were not mature. Furthermore, when EC spheroids were formed, β-cell functional proteins glucose transporter 1 (GLUT1) and proprotein convertase 1, β-cell associated functional genes, and transcription factors were enhanced [22]. The continuous exposure of ALK5iII, T3, and BMP inhibitor with the inclusion of a notch inhibitor (gamma secretase inhibitor XX) resulted in a PDX1-positive/NKX6.1-positive/NEUROD1-positive cell population in which only insulin was expressed [23].

Resveratrol (3,5,4′-trihydroxytrans-stilbene) is a polyphenol compound which stimulates sirtuins1 (SIRT1), a NAD-positive dependent deacetylase [102]. SIRT1 plays an essential role in maturation, as well as in the early differentiation processes [103]. Vetteli et al. studied the effect of resveratrol on insulin secretion using human islets and INS1E cell line [104]. The transcription of mitochondrial uncoupling protein 2 (UCP2) can be achieved through SIRT1, where it straightforwardly binds to the active site of UCP2, as demonstrated by Bordone et al. [105]. SIRT1 overexpression lowers the UCP2 level, resulting in elevated insulin secretion and ATP production [64]. Another target of SIRT1 and resveratrol is transcriptional cofactor peroxisome proliferator-activated receptor-g coactivator1 a, which activates UCP2 in humans [106]. Therefore, resveratrol is the best option for regulating the maturation of insulin-secreting cells.

Important pancreatic lineages can be derived from multipotent progenitors, e.g., Acinar, ductal, and endocrine cells [107,108,109,110,111,112]. Progenitor cells are apparent and proliferative, and with the passage of time, they remain the same after transplantation [113,114]. Small molecules and growth factors are used to manage the proper attachment of the progenitor cells to the feeder layer to ensure their generation and maintenance (RA, notch inhibitor, EGF, TGF-β inhibitor, and FGF10). The number of PDX1-positive cells remains same after blocking HES1 in hESCs, while notch pathway-related studies have not been thoroughly undertaken on humans [41]. Cells treated with factors including Betacellulin, Vitamin E, Heparin, ALK5iII T3, TGF-β, XXI (Gamma secretase inhibitor of notch signaling), N-acetyl cysteine, and AXL receptor tyrosine kinase inhibitor promote nuclear localization and the production of MAFA [17,23,25].

## 5. Efficacy of Small Molecules-Induced Insulin Producing β-Like Cells

The treatment of stem cells (hPSCs, hMSCs) with different factors (cytokines/small molecules) leads to the development of pancreatic β-like cells in a stepwise manner. Stem cell (ESC/ iPSC/MSC) -based protocols take the least amount of time (1–3 weeks) for pancreatic β-like cell generation in vitro [19,65]. Almost all researchers have used high glucose and no/low serum concentrations in the culture to stimulate endocrine β-like cell differentiation, although the mechanism of action for this is still unknown. In addition, various protocols have shown minimal expression of the main β-cell markers, PDX1 and NKX6.1 [15,17]. Among the many studies, very few have achieved glucose-stimulated insulin secretion (GSIS) from stem cell-differentiated pancreatic β-cells in vitro and in vivo (Table 1) [15,17,22,24]. In addition, many studies have failed to demonstrate GSIS and glucose homeostasis restoration after the transplantation of differentiated insulin-producing cells [115]. Pancreatic marker expression (α, β, and δ genes expressed together in the same cell) varies among the various studies [116].

In 2016, Kim et al. developed islet like structures with insulin-producing β-like cells in which ECCs enhanced insulin secretion in response to glucose stimulus, and under transplantation, ß cell-deficient mice survived for more than 40 days and maintained blood glucose levels to some extent [22]. In this study, PDX1 expression was reduced in the transplanted tissues but was observed in the cytoplasm of transplanted tissue. The mice were sacrificed 13 days after transplantation. As 3D microenvironments are necessary for the generation of pancreatic endocrine cells, some researchers have used a combination of matrigel and collagen to create better niches for the efficient differentiation of pancreatic β-like cells, in which mature β-cell markers e.g., PDX1, NGN3, MAFA, GLUT2 and C-peptide expression were observed [117]. Embryoid bodies derived from hPSCs were cultured with endothelial cells in collagen gels in order to provide an appropriate microenvironment, resulting in higher expression of mature β-cell markers with enhanced C-peptide and insulin secretion in vitro and in vivo. In addition, negligible expression of glucagon and somatostatin was observed [118]. In 2014, Melton et al. observed that monohormonal pancreatic β-like cells and polyhormonal cells both express C-peptide-positive/INS-positive cells, but that polyhormonal cells don’t mimic the adult islet INS-positive cells, while monohormonal cells more closely resemble those of native islet β-cells. Also, monohormonal cells were able to respond to GSIS, and the transplantation of these cells was shown to ameliorate high blood glucose levels in diabetic mice [17]. Furthermore, some researchers generated monohormonal cells, but were not able to express mature β-cell markers nor glucose homeostasis restoration after transplantation in vivo [115]. This may be due to the insufficient maturation of the differentiated cells, as the monohormonal cells generated by Melton group were functionally active in vitro and in vivo, as described above. Moreover, Rezania et al. also examined monohormonal β-cells which were not able to respond glucose. Finally, mature β-like cells with the efficient ability to restore glucose homeostasis in vitro as well as in vivo were produced [23]. While some studies have suggested that the coexpression of α- and δ-cells may be critical to mimicking native β-cells [119,120], glucagon and somatostatin secretion may be crucial in the treatment of noninsulin-dependent DM, as both hormones appear to control insulin release in a paracrine manner [116].

Evolution from research to preclinical studies, and lastly, to clinical trials is occurring in many fields (Table 4). Some researchers have administered a WJ-MSC infusion to T2DM patients, resulting in the improvement of β-cell function and reducing the severity of complications experienced by diabetic patients [121].

## 6. Reprogramming

One substitutive method involves reprogramming terminally-differentiated cells into pancreatic β-like cells from stem cells in a stepwise manner. This requires the overexpression of two or more genes. Previously, researchers have developed a direct lineage-specific reprogramming strategy by which other cell types can be reprogrammed into pancreatic β-like cells [122,123,124,125]. 

### 6.1. Reprogramming of Pancreatic Exocrine Cells into β-Like Cells

A cre-mediated recombination of β-cell-specific factors (Sox9-CreER; CAG-CAT-Glp1r) in SOX9 (Sex-determining region Y-box 9) expressing cells was treated with Exendin-4 alone or in combination with Gastrin, resulting in the overexpression of GLP1R in exocrine cells. Therefore, β-like cell can be induced from exocrine cells by the activation of GLP1 and Gastrin signaling without any ill effects [68].

### 6.2. Reprogramming of Acinar and Duct Cells

Partial duct ligation negates boost islet mass, apparently through the transdifferentiation of surrounding tissues, where the formation and depletion of ductal and acinar cells occur simultaneously [126]. Xu and colleagues demonstrated that NGN3 reexpression can be induced by partial duct ligation, and that adult EPs may be transformable into new β-like cells [127]. However, it was revealed that partial duct ligation doesn’t influence the β-cell number, so the increased β-cell numbers could be an artefact [128,129]. After partial duct ligation, PTF1A-positive acinar cells could transdifferentiate into NGN3-positive progenitors [110]. Insulin-secreting cells differentiated from PTF1A-positive acinar and reprogrammed acinar cells, with both showing similar expression of the mature pancreatic β-cells markers NKX6.1, PDX1, and MAFA [110,125]. The effect of hyperglycemia on reprogramming in vitro is still unknown. In 2015, Cavelti-Weder et al. found that hyperglycemia negatively affects exocrine cells in terms of insulin-positive cell reprogramming in vitro [130]. Many reprogrammed cells could be obtained when such studies were performed in a normal glycemic environment, as compared to a hyperglycemic one.

#### 6.2.1. Acinar Cells to Pancreatic β-Like Cell Induction

Acinar cells have potential to be reprogrammed into β-like cells, as they have a similar developmental origin [125,131]. Exocrine acinar cells are highly differentiated and maintain a significant degree of plasticity. Acinar cells can be reprogrammed to β-like cells by the ectopic expression of pancreas-specific transcription factors; these factors lead to the reprogramming of acinar cells into β-like cells, such as the three reprogramming factors, NGN3, MAFA and PDX1, referred to as M3 factors. Initially, researchers checked various transcription factors that were present in pancreatic β-cells and their precursors, but they concluded that PDX1, NGN3, and MAFA were sufficient for reprogramming to generate β-like cells [125]. Humoral factors that regulate pancreatic development include GLP1, Exendin-4, Incretins, and growth factors [21,132]. In vitro lineage tracing revealed the transformation of CPA1-positive mature acinar cells into insulin-producing cells. Importantly, with the loss of acinar cells, the coexpression of specific markers PTF1A or amylase, NKX6.1, GCK, and GLUT2 (Slc2a2) was observed. Finally, glycemic control in diabetic mice can be controlled sufficiently by induced cells; this is a key feature of native β-cells [125]. The reprogramming of acinar to β-like cells yields insulin-positive cells which remain functional for at least 13 months [133]. In 2009, Baeyens et al. demonstrated that the transformation of adult acinar cells into β-like cells is inhibited by notch signaling. The reexpression of NGN3 regulates the embryonic endocrine pancreatic development. The down-regulation of notch signaling leads to the conversion of acinar cells in response to growth factor-based differentiation. Approximately 30% of acinar cells could be converted into β-like cells through notch inhibition. When newly-formed, mature β-like cells were ectopically transplanted, they were able to control glycemia in diabetic patients [134]. Signal transducers and activators of transcription 3 signaling and Mitogen-activated protein kinase are known effectors of EGF and LIF (Leukemia inhibitory factor) which control the dedifferentiation of acinar cells into endocrine cells [134].

#### 6.2.2. Duct Cells Reprogrammed into Pancreatic β-Like Cell

Duct cells can also be reprogrammed by the adenoviral transduction of NGN3 transcription factor to express pancreatic β-like cell genes. For the full reprogramming of duct cells to endocrine cells, NGN3 is not sufficient. So, reprogramming is improved by the down-regulation of delta-notch signaling [135] and the coexpression of MYT1 [136], MAFA, and PDX1 [125] pancreatic endocrine factors [137]. The transduction of NGN3 transcription factor promotes the auto-activation of this gene endogenously. Islet endocrine cell differentiation from epithelial progenitors is initiated by NGN3 activation [107]. The proteolytic step of the bioactive notch domain is blocked by gamma-secretase inhibitor (L685, 458). The gamma-secretase inhibitor enhances the endogenous secretion of NGN3 without affecting transduced duct cells, ultimately leading to insulin expression.

#### 6.3. α-Cell to β-Like Cell Reprogramming

α-cells may have some β-cell-specific genes that are suitable for reprogramming into β-like cells. Histone methyl-transferase inhibitor has partial transcriptional potential to express PDX1 and insulin in glucagon-positive cells when islets are treated with it, which is an epigenetic kind of reprogramming [138,139]. It was hypothesized that in the absence of β-cells, some transplanted hPSC-derived α-cells could be directly converted into insulin-producing cells in vitro. Thorel and colleagues made a transgenic model using a diphtheria toxin receptor system which made possible α-cell lineage tracing and complete β-cell removal [140]. Total β-cell ablation led to the consequent regeneration of β-cells, but many of these cells were derived from former α-cells.

## 7. Ectopic Expression

Ectopic expression is a unique and useful technique, where the cell is not able to express genes normally. Compared with basal expressions, the function of the gene of interest can be identified [141]. Even though ectopic expression deals with endogenous genes in an organism like transgenesis; exogenous gene is introduced into a cell or tissue, where the gene itself is not usually expressed [142]. This practice is not only limited to the identification of gene functions in a known cell, but can also be used to reveal unknown functions of genes.

### 7.1. Ectopic Expression of PAX4

The PAX4 ectopic expression to α-cells leads to the induction of β-like cells in vitro [131,143]. In addition, PAX4 loss is directly related to the destruction of β-cells and an enhanced number of α-cells [144]. The ectopic expression of PAX4 in δ-cells results in the conversion and regeneration of pancreatic α-cells into β-like cells. Researchers developed and identified animal models which expressed PAX4 in somatostatin-expressing cells in order to check the plasticity of δ-cells. It was shown that PAX4 ectopic expression is sufficient for the conversion of δ-cells into functional insulin-producing cells. Mostly, these induced insulin-producing cells were functional and could slightly change the consequences of chemically-induced diabetes [145]. The ectopic expression of PAX4, either in PDX1-positive or PAX6-positive endocrine cells, led to the observation of β-cell characteristics. PAX4 acts on the specification of EP cells by enhancing the pancreatic β-cell lineage [131,145]. It was shown that the specific loss of ARX and PAX4 ectopic expression in α-cells leads to the transformation of α-cells into insulin-producing cells [63,131,146]. The haematopoietically-expressed homeobox (Hhex) transcription factor has been repeatedly linked to T2DM in genome-wide association studies, and is needed for the specification of δ-cell [147] and PAX4 ectopic expression results in the suppression of Hhex. It also promotes pancreatic cell lineage. All these findings therefore allow us to conclude that the expression of PAX4 in somatostatin-positive cells promotes their differentiation into β-like cells, apparently through Hhex down-regulation [145].

### 7.2. Ectopic Expression of GIP

Pancreatic α-cells manage the production of glucagon and GLP1, respectively, and play important roles in glucose homeostasis [148]. The glucagon gene encodes proglucagon, GLP1, oxyntomodulin, and GLP2 [149]. The main role of glucagon is to regulate the production of glucose in the liver by stimulating glycogenolysis and gluconeogenesis; in contrast, in response to hypoglycemia, it leads to the inhibition of glycogen synthesis and glycolysis [148]. Any impairment of glucagon secretion aggravates DM through the increased production of hepatic glucose [150]. Glucagon is crucial in the regulation glucose homeostasis as a hyperglycemic hormone, as demonstrated by interfering with glucagon signaling in transgenic mouse models [151,152,153]. The down-regulation of glucagon signaling was shown to increase glucagon and GLP1 levels. The increased GLP1 level resulted in improvements of pancreatic β-like cell function. Increased insulin secretion in glucagon-deficient mice was improved by blocking glucose-dependent insulinotropic polypeptide (GIP) in vivo and in vitro by cAMP antagonism and the deletion of the GIP receptor, respectively [154]. The activation of Protein kinase A could be blocked by Rp-cAMP (a cAMP antagonist), as intracellular cAMP signaling mostly regulates GIP signaling [155].

The ectopic expression of GIP in β-like cells regulates the secretion of insulin when proglucagon-derived peptides are not present. One significant approach to treating T2DM is the inhibition of glucagon function [148,156]. Glucagon plays an essential role in the regulation of glucose homeostasis, which is indicated by suppressing glucagon signaling in genetically-modified organisms [151,152,153].

It was demonstrated that the removal of proglucagon-derived peptides led to increased β-like cell function that could be connected to the induction of GIP expression in β-like cells. It was therefore concluded that GIP is crucial in the regulation of insulin secretion [157].

### 7.3. Ectopic Expression of PDX1

Adenovirus-mediated PDX1 gene transfer into the adult liver induces both exocrine and endocrine pancreatic gene expression [158,159,160]. The activated version of PDX1 (PDX1-VP16), under the control of the liver-specific transthyretin promoter, is sufficient for the transformation of liver to pancreas [161].

In 2003, Ritz-Laser et al. showed that PDX1 was about to inhibit the glucagon gene in glucagonoma cells [162,163,164]. While PDX1 induction leads to the expression of the islet-amyloid-polypeptide and insulin gene, no effect was observed on the expression of glucagon gene [165].

However, these studies were not able to differentiate between the direct and indirect impact of PDX1 on glucagon and insulin gene expression, respectively. When PDX1 is overexpressed in glucagonoma cells, it plays an essential role in the insulin and glucagon gene expression when other β-like cell-specific factors are not present. It also demonstrated its important function in the differentiation of pancreatic β-like cells. Glucagon gene expression is mediated by the adverse effects of PDX1 in vitro by debilitating the PAX-6- and CDX-2/3-derived activation of the G3 and G1 promoter elements. [166]. Two mechanisms can describe the down-regulation of transcriptional activation by PDX1: (i) direct interactions with PAX-6 and CDX-2/3, PDX1 [167], and (ii) PDX1 may effectively block transcriptional activation by disturbing communication with transcription factors which are bound to DNA. Recently, it was shown that insulin promoter activity could be down-regulated when insulin-producing cells were overexpressed with PDX1 [168].

### 7.4. Ectopic Expression of PDX1 and NKX6.1

NKX6.1 is an essential transcriptional factor for the effective maintenance of mature β-cell function and identity. It is also the master regulator of actions like the direct activation of β-cell functional programs and the down-regulation of non–β cell lineages. NKX6.1 activates NGN3 and ISL1 markers, which are immature pancreatic markers, but it doesn’t express the genes related to pancreatic hormones. The implementation of NKX6.1, together with the ectopic expression of PDX1, was shown to promote the production of insulin compared to the ectopic expression of PDX1 alone, without affecting reprogrammed cells. NKX6.1 is essential in promoting the maturation of PDX1 reprogrammed cells along with β-like cell lineages [169]. Similarly, adult human liver cells can generate insulin-secreting cells in vitro. Reprogrammed human liver cells produce the functional insulin hormone [170].

### 7.5. SIRT5 (Sirtuin 5) Regulates Pancreatic β-Like Cell Proliferation and Insulin Secretion

Sirtuin (SIRT) proteins have nicotinamide adenine dinucleotide-dependent deacetylase activity, which is a part of the histone deacetylase family, consisting of seven subtypes in mammals (SIRT1 to 7) [171]. SIRT5 targets the metabolic enzymes involved in the synthesis of ketone bodies, glycolysis, the tricarboxylic acid cycle, ATP synthesis, amino acid catabolism, and fatty-acid β-oxidation [172]. As SIRT5 controls the expression of metabolic enzymes, it plays a key role in diabetes. However, its practical role in diabetes is still not known. PDX1 protein plays a number of roles in diabetes [173]. It is hypothesized that PDX1 expression may be regulated by SIRT5 in diabetic patients [174]. In order to prove this hypothesis, SIRT5 was knocked in and out in MIN6 and INS1 cells, and the PDX1 expression was analyzed. This means that SIRT5 regulates PDX1 expression. When the expression of SIRT5 was knocked in and out, PDX1 expression was increased and decreased, respectively. The overexpression of SIRT5 deacetylase (T69A) (dormant form) revealed that the dormant PDX1 transcription could be regulated through the deacetylase activity of SIRT5, as its deacetylase activity controls PDX1 expression [174]. Previous studies have demonstrated that histone deacetylase inhibitors VPA and TMP269 enhance PDX1 expression, which ultimately elevates the secretion of insulin in vitro and in vivo. TMP269 also improved the differentiation of endocrine-like cells by increasing the number of insulin-producing cells [175,176]. Finally, it was concluded that the ectopic expression of SIRT5 is negatively related to PDX1 expression, but positively related with glucose level. Additionally, it results in the down-regulation of PDX1 expression, and the dysfunction of SIRT5 leads to the elevated expression of PDX1, which means that SIRT5 plays a significant role in the differentiation of pancreatic β-like cells [174].

### 7.6. Ectopic Expression of PDX1, NGN3, MAFA

PDX1, NGN3, and MAFA are important transcription factors for the development and maturation of β-like cells, and are crucial for the transdifferentiation of acinar to β-like cells [124]. Gene expression may be controlled through transcription factors binding to specific active sites. As discussed earlier, for the development of pancreatic cell lineages, PDX1 expression is necessary. PDX1 binds to the regulatory sites and stimulates transcription of the insulin gene. Similarly, MAFA regulates the function of insulin by binding to the promoter site of the insulin gene. Any other type of cell can be transformed into a functional pancreatic β-like cell by the ectopic expression of PDX1, MAFA, and NEUROG3 in vitro and in vivo [52,177].

## 8. Bio-Engineering and 3D-Printing Technology

Nowadays, bioengineering-based approaches are widely used in the construction of organ/organoids by imitating the native microenvironment. Bio-printing techniques emerged in 1988 [178]. Regenerative medicine and tissue engineering are showing promising outcomes, e.g., the efficient generation of functional bioengineered pancreatic β-like cells. Among bioengineering techniques, decellularization is one where an organ-specific acellular matrix can be generated and allowed to proliferate with cell populations of interest. Biomaterial science is an integrative platform directed towards the biological and physical activities of materials.

### 8.1. Microenvironment Deliberation

Regardless of the impact of ECM and oxygen tension on the survival of islets, the physiological environment is widely neglected in differentiation protocols of β-like cells. Islet cells are intensely sensitive to both high and low concentration of oxygen. At the same time, in order to attain a functional phenotype, they need proper 3D cell-to-cell interaction. Until now, in vitro conditions were not able to mimic proper in vivo environmental conditions. Physiological oxygen circulation is not compatible due to the lack of capillary vessels in the 3D islet-like structure. For novel differentiation, both oxygen circulation and ECM are crucial for reconstructing pancreatic β-like cells during differentiation. So, ECM components like laminins, collagen, silicone rubber, and fibronectin membranes have been used to control oxygen tension [179,180,181]. Recently, pancreatic tissue derived ECM (pdECM) was used to recreate in vivo environment conditions to differentiate functional pancreatic ECs [181].

### 8.2. Engineering a 3D Niche for Pancreatic Cells

Generally, pancreatic β-like cells are encircled with pancreas-specific ECM proteins, consisting mainly of a basement membrane and interstitial matrix proteins like laminin, collagen type IV, and fibronectin. The matrix plays a major role in islet function, proliferation, and survival [180]. Fundamental requirements for cellular transplantation include porosity, biocompatibility, the surface area to volume ratio, and appropriate culture conditions for the development of newly-formed cells [182]. The porous property of the scaffold is an important factor for cellular energy, the proper distribution of nutrients, and oxygen to cells. Pores with a diameter under or over 50µm are characteristic of micro- and macro- porous synthetic scaffolds, respectively [183]. Chitosan [184] and polylactic-co-glycolic acid are important synthetic polymers [185]. Insulin secretion and the maturation of cells are highly up-regulated when insulin-producing cells are cultured with pdECM [181].

### 8.3. ECM Scaffold

Improvement and protection of impaired pancreatic function can be achieved by tissue engineering. To that end, 3D ECM is relatively a new technology which is being tested to make scaffolds based on ECM that can be multiplied with autologous cells of patients. The process by which cellular components from a tissue or organ are removed while retaining the actual structure and composition of the associated ECMis known as decellularization. This process maintains the biochemical signals of ECM as well as the micro/macro structure of the tissue/organ [186]. Poly-L-lactic acid and polyvinyl alcohol scaffolds lead to maintenance of the microenvironment, metabolic activity, and the expression of the transcription factors necessary for the differentiation of pancreatic β-like cells [187]. In 2018, Mansour et al. manufactured polyethersulfone nanofibers by electrospinning, and then coated them with collagen, as this is abundantly present in pancreatic ECM. When iPSCs were cultured on polyethersulfone-collagen, pancreatic proteins/markers were highly expressed compared to those of the 2D control group. Concerning tissue engineering applications, polyethersulfone-collagen could be an appropriate candidate to differentiate pancreatic β-like cells [188].

The growth factors secreted by specific ECM should be as good as those of the native microenvironment for the proper growth of cells. Researchers have hypothesized that the pancreatic structure will be a better platform for pancreatic β-like cells. They therefore attempted to prepare mouse/porcine-derived pancreatic β-like cells using a decellularization technique. For the clinical utility of scaffolds, these were tested for pancreatic tissue bioengineering on porcine models [189]. In 2019, Kim et al. successfully developed pdECM bioink from decellularized porcine pancreas by treating it with a number of chemicals, followed by lyophilisation and dissolving the powder. Functional insulin-producing cells were derived by improving the microenvironment conditions [181].

### 8.4. Vascularization and Encapsulation Technology

Wrapping islet cells with a biocompatible membrane allows the proper distribution of nutrients to occur, as well as protecting islets from bigger molecules like immune cells and antibodies. There are several methods of encapsulating cells with hydrogels like polysulfone, PEG (Polyethylene glycol), PLL (Poly-l-lysine), and alginate, under 3D culture conditions [190,191,192]. Due to poor or reduced biocompatibility and low structural stability, the function and viability of encapsulated islet cells decrease [193]. The PEC-Direct™ device makes it possible to directly vascularize specific PPs, assuring their differentiation and transformation into pancreatic β-like cells. One of the main problems may be direct contact between the host and the transplanted cells, necessitating immunosuppressive treatment [100,194]. These limitations could be overcome by using a fine porous coating which allows proper dispersion and vascularization to occur in order to prevent direct contact between the host and the transplanted cells, and ultimately, to prevent life-long immunosuppression (Via Cyte).

Microporous scaffolds have also shown potential as a bio-manufacturing platform to generate insulin-producing β-like cells [73,195,196,197]. Moreover, 3D culture has a higher expression of insulin than 2D culture. This suggests that pancreatic scaffolds augment the generation of pancreatic β-like cells derived from iPSCs, and are powerful therapeutic means to cure DM [181,198]. After transplanting encapsulated islets with degradable PEG hydrogel, by the third week of transplantation, constant normoglycemia was observed, and it was concluded that the microporous hydrogel quickly maintained glucose levels in blood [199,200].

### 8.5. Amikagel Based Platform

Amikagel is a novel hydrogel system which expedites the controlled and impulsive aggregation of hESC-derived PPs into influential spheroids [201]. Compared the gold standard, Matrigel, the islet phenotype could be elevated by the incorporation of Amikagel. It induces both an arrangement of 3D spheroids and the expression of pancreatic markers, PDX1 and NKX6.1. Additionally, the NKX6.1 and PDX1 coexpressing cell populations are elevated, demonstrating a more suitable population for islet maturation. Amikagel also permits the coaggregation of hESC-PPs and endothelial cells to occur, by which pancreatic organoids of similar islet physiologies are formed in vitro [202]. Amikagel-induced heterogeneous organoids and PPs spheroids could additionally be differentiated into insulin-producing β-like cells which express INS1 as well as C-peptide protein. These organoids transform into β-like cells without specific induction using chemicals, and show mature islet function, i.e., GSIS in vitro. Thus, the use of Amikagel opens up opportunities for the construction of islet organoids. The next generation of vascularized human organoids could be optimized using Amikagel for applications in regenerative medicine and drug discovery.

### 8.6. Organ on Chip

A new strategy has been devised to engineer hiPSC-derived pancreatic cells using an organ on a chip platform. The islet on a chip system is robust and amenable for islet organoid growth; it serve as a suitable method for engineering organoids, disease modeling, and regenerative medicine [203]. This technique generates functional insulin-producing cells, integrates regulated EBs formation, and generates and differentiates islet organoids on a single chip for a longer period with enhanced function and survival of the islets [203].

## 9. Endothelial Cell Coculture

As discussed earlier, endothelial cell signaling is crucial to control PDX1 expression; this is also the reason for SHH inhibition. A few researchers have cocultured endothelial cells, leading to the appropriate assimilation between blood vessels and islets. The endothelial cells signaling effect is progressively being acknowledged as a significant contributing factor in the maturation of pancreatic islet in vitro. Recently, the endothelial cells signaling effect was shown in the production of human PPs into insulin-secreting cells. As a result, glucose homeostasis, as well as increased insulin production and secretion ability, are achieved, which indicates the efficiency of these techniques in the promotion of therapeutic outcomes [85,204,205,206]. In recent research, to set up a vascular network in vitro endothelial cells and human islets were cocultured; it was concluded that proper contact between islet and endothelial cell is essential for islet function [180].

## 10. Expected Outcomes

Regarding the aforementioned literature and the merits and shortcomings of the presently available protocols, it has been found that many modifications are still needed before this approach can be applied in clinical settings. We have tried to discuss almost all of the latest updates regarding various approaches to differentiate pancreatic β-like cells from stem cells.

1) Development of cheaper, more efficient, and faster protocols for the pancreatic lineage.
2) Resolution of microenvironmental issues by using bio-scaffolds and bio-printing.
3) A homogenous or pure population of pancreatic β-like cells is expected to be obtained.
4) Attainment of an increased number of insulin-positive cells with long-term in vitro and in vivo viability.
5) Development of highly potent pancreatic β-like cells in vitro with the ability to be preserved for longer durations without the loss of cellular and functional properties.
6) Preclinical studies need to follow, including investigations of blood glucose regulation after transplantation and immune protection methods.
7) Finally, achieving a life-long cure for diabetes by utilizing differentiated/reprogrammed/bioengineered stem cells without causing any ill effects.

## 11. Conclusions

This study discusses the generation of functionally active insulin-producing cells which have similar survival rates to those of native β-cells, as well as GSIS ability, upon transplantation into experimental models. β-cell differentiation/islet organoid formation approaches are regulated by a complex system that relies upon the transcriptional management of genes which are associated with pancreatic development, specific types of differentiation factors (small molecules, cytokines, and growth factors), and culture conditions. Even though various aspects are crucial for the satisfactory formation and transplantation of insulin-producing β-like cells or organoids, proper cell culture conditions and the mixture of specific small molecules stand out as the most important factors. Still, more effort is required to enhance the functional maturity and survival of stem cell-derived insulin-secreting cells or islet-like organoids after transplantation, which can mimic native pancreatic islets/β-cells. Once insulin-secreting cells have been generated, it is necessary to think about storage strategies. In future, these cells can be used to treat diabetic patients. In short, we also need to pay attention to the longevity and storage of stem cell-derived pancreatic β-like cells/islet organoids.

## Figures and Tables

**Table 1:** Overview of small molecules involved in the development process and showing the functionality of β-like cell-derived from various sources of stem cells.

Stem Cell Source	Protocol	DE Markers (%)	PE Markers PDX^+^ (%)	PPs Markers (%)	Insulin Producing Cell INS^+^/C- Peptide^+^/GCG^+^ (%)	In vivo Efficacy: Glucose Homeostasis Restoration	GSIS	References
hESCs	Day 1–2: Activin A+WNT3A. Day 3–5: TGF-βi+KGF. Day 6–8: Noggin+RA+Cyclopamine. Day 9–10: EGF+KGF+Noggin. Day 11–20: TBP+ALKi+Noggin	NA	> 88% PDX1^+^	80% NKX6.1^+^/PDX1^+^	92% NKX6.1^+^/C-peptide^+^	√	In vitro √; In vivo √	[15]
hESCs, hiPSCs	Day 1: Activin A+CHIR. Day 2: Activin A. Day 4–6: KGF. Day 7–8: KGF+SANT1+RA+LDN+PdBU. Days 9–13: KGF+SANT1 +RA. Day 14–16: SANT1+RA+XXI+ ALK5iII+T3+Betacellulin. Day 18–20: RA+ XXI+ALK5iII+T3+Betacellulin. Day 21–35: ALK5iII+T3	>95% SOX17^+^	>85% PDX1^+^	>55% NKX6.1^+^/PDX1^+^	8% C-peptide^+^/GCG^+^	√	In vitro √; In vivo √	[17]
hiPSCs	Day 1–3: Activin A+CHIR+WNT3A. Day 4–10: Noggin+Dorsomorphin+RA+SB431542. Day 11–21: Forskolin+Dexamethasone+ALK5iII+Nicotinamid.	75% SOX17^+^/ FOXA2^+^	NA	72% PDX1^+^	8–16% INS^+^	NA	In vitro √	[19]
hESCs	Day 1: Activin A+Li+CHIR. Day 2–5: Activin A. Day 6–11: RA+Dorsomorphin+SB431542+KAAD-Cyclopamine+FGF2. Day 12–15: DAPT+Dorsomorphin+SB431542 +Ascorbic acid. Day 16–23: Db-cAMP+Exendin-4+Dorsomorphin+SB431542+Nicotinamide+Ascorbic acid	>94% CXCR4^+^	93% PDX1^+^	NA	NA	√	In vitro √; In vivo √	[22]
hESCs	Day 1–3: GDF8+GSK3-βi. Day 4–5: FGF7+VitC. Day 6–10: FGF7+VitC+RA+ SANT+TPB+LDN. Day 11–13: SANT+RA+ ALK5iII+T3+LDN. Day 14–28: ALK5iII +T3+LDN+GSiXX. Days 28–43: ALK5iII +T3+N-Cys+AXLi.	NA	NA	76%	31–38% NKX6.1^+^/INS^+^ 21% NKX6.1^+^/GCG^+^	√	In vivo √	[23]
hiPSCs	Day 1–2: CHIR+FGF2+Activin-A+BMP4. Day 3–5: FGF2+Activin-A+BMP4. Day 6–7: FGF2+FGF7+EC23+SB431542+Dorsomorhin+SANT1. Day 8–11: FGF2+EC23+SB431542+ Dorsomorphin+SANT1. Day 12–14: FGF10 +EC23 +Dorsomorphin+SANT1+ALK5iII +ILV. Day 15–17: EC23+Dorsomorphin +SANT1+ALK5iII+Exendin-4. Day 18–23: BMP4+FGF2+HGF+IGF+Nicotinamide+Forskolin+Exendin-4+ALK5iII	>80% SOX17^+^; >68% FOXA2^+^	92% PDX1^+^	NA	34% C-peptide^+^	√	In vitro √; In vivo √	[24]
hiPSCs	Day 1–3: Activin-A+CHIR. Day 4–6: KGF. Day 7–8: KGF+RA+SANT1+Y27632+LDN+PdbU. Day 9–13: KGF+RA+SANT1 +Y27632+Activin A. Day 14–20: RA+SANT1+T3+XXI+ALK5i+Heparin+Betacellulin. Day 21–34: T3+ALK5i+ CMRL supplemented	NA	NA	52-89% NKX6.1^+^/PDX1^+^	30% NKX6.1^+^/C-peptide^+^	√	In vitro √; In vivo √	[43]
BM-MSCs	Day 1–2: β-ME. Day 3–10: NEAA+bFGF+EGF+2% B27+L-glutamine. Day 11–18: Betacellulin+Activin-A+2% B27+Nicotinamide.	NA	NA	NA	5–10% INS^+^/C- peptide^+^	√	In vitro √; In vivo √	[44]
ASCs	Day 1–3: Activin-A+Sodium butyrate+ITS+β-ME. Day 4–5: Taurine+ITS. Day 6–10: Taurine+ITS+Nicotinamide+NEAA+GLP-1.	28% SOX17^+^; 22% FOXA2^+^	65% PDX1^+^		48% C-peptide^+^	√	In vitro √; In vivo √	[45]
hMSCs	Day 1–7: Nicotinamide. Day 8–14: Exendin-4	NA	NA	NA	15% INS^+^ 6% C-peptide^+^	√	In vitro √; In vivo √	[46]

√ (Yes): Indicates the efficiency of differentiated cells towards Glucose Homeostasis Restoration and GSIS.

**Table 2:** Role of different transcriptional factors in pancreatic endocrine cell development.

Stage	Transcriptional Factors	Function
DE	SRY-related HMG-box 17	Regulation of embryonic development
	Forkheadbox A2 (HNF-3β)	Endodermal marker, differentiation of pancreas
	C-X-C chemokine receptor type 4	Chemokine signals in early pancreatic differentiation
	Cerberus	Endodermal marker, differentiation of pancreas
	Transcription factor GATA-4	Regulates the development of endoderm-derived organs
	Brachyury protein	Brachyury is an important factor in promoting theepithelial-mesenchymal transition
	Orthodenticle homeobox 2	Influences proliferation and differentiation
	Homeobox protein goosecoid	Cell-fate specification
	Receptor tyrosine kinase	Involved in intracellular signaling
	Mix paired-like homeobox	Plays a role in proper axial mesendoderm morphogenesis and endoderm formation
	Hepatocyte nuclear factor 4 α	Controls the expression of the FOXA2 and SOX17 genes
PGT	Pancreatic and duodenal homeobox	Early pancreatic development, α- and β-cell, and exocrine tissue genesis, important activator of insulin
	Transcription factor SOX-9	Regulates epithelial progenitor expansion and endocrine differentiation
	Hepatocyte nuclear factor 1 homeobox B	Plays a crucial role in early development
PE	NK6 TF related locus 6	Final differentiation of β-cells
	Homeobox protein CDX-2	Tumor suppressor in pancreas
	Sex determining region Y	Essential for maintaining self-renewal and pluripotency
	Motor neuron and pancreas homeobox 1	Regulation of β-cell development
PPs	NK2 TF related locus 2	Pancreatic endocrine development and differentiation into pancreatic β-cells
	Neurogenin 3	Formation of pancreatic endocrine precursors, differentiation of pancreatic precursor cells towards endocrine lineage
	Neurogenic differentiation	Differentiation and islet growth, endocrine differentiation in pancreatic progenitors
	Paired box gene 6	Formation of α-cells, activates glucagon transcription
	Paired box gene 4	Formation of β-cells and δ-cells, repress glucagon transcription
	Hepatocyte nuclear factor 6	Essential for endocrine differentiation
	Islet 1	Early endocrine cell differentiation
	Pancreas associated transcription factor 1	Stage-specific roles during pancreatic organogenesis
EPs	Avian musculoaponeurotic fibrosarcoma oncogene family A	Formation of α- and β-cells, activates genes involved on mature endocrine functions like glucose sensing, vesicle maturation, calcium signaling, and insulin secretion
	Avian musculoaponeurotic fibrosarcoma oncogene family B	Controls and activates insulin gene expression
	Chromogranin A	Maintains islet volume, cellular composition, and function
	Insulin	Pancreatic β-cell maturation
	Glucagon	Produced by pancreatic α -cells, leads to increased gluconeogenesis
	Somatostatin	Regulates the endocrine system
	Ghrelin	Regulates homeostasis
	Pancreatic polypeptide	Metabolic homeostasis

**Table 3:** Small molecules involved in various stages of β-cell differentiation from stem cells.

Small Molecules	Function	Application	Stem Cell Sources	References
IDE1/2	Activator of TGF-β pathway	Induces DE formation	hPSCs	[59,60]
NECA	Adenosine receptor agonist	Promotes β-cell proliferation	Fibroblasts	[41]
Dexamethasone	Agonist of glucocorticoid receptor	Enhances β-cell proliferation	hESCs, hiPSCs	[19,61]
ALK5iII	ALK5 inhibitor	Promotes β-cell differentiation and maturation	hESCs, hiPSCs	[15,17,19,41,61,62,63]
Taurine	Alter membrane potential and have an effect on ion currents	Secretion of insulin	hESCs	[40]
SCG (sodium cromoglicate)	Anti-inflammatory	Facilitates the differentiation of PDX1-positive cells into INS-positive cells	hESCs, hiPSCs	[64]
N-acetyl cysteine	Antioxidant	Improves insulin production and secretion	hESCs	[41,63]
Forskolin	AXL inhibitor/ cAMP signaling activator(an activator of adenylyl cyclase)	Promotes β-cell formation	hESCs, hiPSCs	[19,41,61,62]
Noggin	BMP inhibitor	Suppresses hepatic lineage differentiation	hESCs, hiPSCs	[15,18,19,40,59,61,65,66]
Dorsomorphin		Suppresses hepatic lineage differentiation	hESCs, hiPSCs	[19,22,61]
LDN		Promotes pancreatic specification	hESCs	[15,17,25,62,67]
Vit C	Cofactor of epigenetic modulators	Enhances reprogramming efficiency and promotes pancreatic specification	hESCs	[23,62]
RG	DNA methylase inhibitor	Epigenetic modulators	Fibroblasts	[41]
Betacellulin	EGF	Growth and differentiation of β-cells	hESCs, hiPSCs	[17]
DAPT	Gamma secretase inhibitor	Block notch signaling/support long term self- renewal	hESCs, hiPSCs	[22]
CHIR99021	GSK-3β inhibitor	Induces DE formation	hPSCs, hiPSCs	[17,19,22,25,59,61,62,67,68]
Par	Histone demethylase inhibitor	Epigenetic modulators	Fibroblasts	[55]
Sodium Butyrate	Inhibitor of histone deacetylation	Activates genes of early pancreatic development	hMSCs, hESCs, ASCs	[40,69]
Stauprimide	Inhibitor of NME	Destabilizes c-myc pluripotency marker	ESCs	[70,71]
Db-cAMP	Nerve growth factor	Increases insulin secretion by increasing mRNA	hESCs, hiPSCs	[22]
Exendin-4	Peptide analog of GLP1	Improves glucose tolerance by increasing insulin secretion	hESCs, hiPSCs	[22,40,62,72]
Wortmannin	PI3K inhibitor	Enhances yield of DE cells	hPSCs	[61,73]
ILV	PKC activator	Promotes PDX-positive cells	hESCs, hiPSCs	[39,59,74]
PdBU		Promotes pancreatic differentiation	hESCs, hiPSCs	[17,25,67]
TPB		Promotes pancreatic specification	hESCs	[15,41,59]
Resveratrol	Polyphenolic compound	Up-regulation of key genes for β-cell function	hESCs	[75]
Fasudil	ROCK1 inhibitor	Directs pancreatic lineage differentiation	ESCs	[30]
RKI-1447		Directs pancreatic lineage differentiation	ESCs	[30]
Thiazovivin		Directs pancreatic lineage differentiation	ESCs	[30]
Y27632		Pancreatic differentiation	hESCs, hiPSCs	[25,67]
CYC	SHH inhibitor	Promotes pancreatic lineage	hESCs, hiPSCs	[22,59,62,72,75]
SANT-1		Promotes pancreatic specification	hESCs, hiPSCs	[17,19,30,41]
SB171542	TGF-β type 1 receptor inhibitor	Induces NGN3 expression	hESCs, hiPSCs	[19,22,59,62]
T3	Thyroid hormone	Promotes β-cell differentiation and maturation	hESCs	[17,41,63]
Nicotinamide	Vitamin	Promotes generation of progenitors	hESCs, hiPSCs	[22,61]

**Table 4:** Description about the list of clinical trials taken from https://www.clinicaltrials.gov/.

PathologicalCondition	Enrolled Patients	Intervention	National Clinical Trial Number	Outcome Measures	Phase	Status
Autologous mesenchymal stromal cell	24	Autologous mesenchymal stromal cell	NCT02384018	C-peptide level, liver function, kidney function, absence of severe hypoglycemic episodes	Phase 1	Ongoing
T2DM With Renal Manifestations	54	Human umbilical cord mesenchymal stem cells	NCT04216849	Estimated glomerular filtration rate, urinary albumin creatinine ratio	Phase 2	Ongoing
Diabetic Nephropathy	15	Human umbilical cord mesenchymal stem cells	NCT04125329	Incidence of treatment-emergent, treatment-chronic adverse events and estimated glomerular filtration rate	Early Phase 1	Ongoing
Diabetic Nephropathies	20	Wharton Jelly Mesenchymal stem cells	NCT03288571	Incidence of treatment-emergent adverse events, glomerular filtration rate, and protein to creatinine ratio	Phase 2	Ongoing
Diabetic Kidney Disease	48	Mesenchymal Stromal Cells	NCT02585622	Adverse events, glomerular filtration rate, urinary albumin/creatinine ratio, urinary albumin excretion, fasting blood glucose, and fasting blood glucose	Phase 2	Ongoing
T1DM	20	Intravenous Injection of autologous mesenchymal stem cells	NCT04078308	Baseline fasting blood sugar (FBS), assessing fasting blood sugar (FBS), C-peptide concentration, and Insulin uptake	Phase 2	Ongoing
T2DM	30	Expanded autologous bone marrow-derived mesenchymal stem cell	NCT03343782	Insulin dose, adverse events, and hemoglobin A1c (HbA1c) level	Phase 2	Completed
T1DM	20	Mesenchymal stem cells	NCT01068951	C-peptide concentration	NA	Completed
T2DM	200	Umbilical cord mesenchymal stem cells	NCT02302599	Change from baseline in fasting glucose over time	Phase 1	Completed

## Abbreviations

2D	Two dimensional
3D	Three dimensional
ALK5iII	Activin receptor-like kinase 5 inhibitor II
ASCs	Adult stem cells
bFGF	Basic fibroblast growth factors
BMP	Bone morphogenic protein
DAPT	N-[N- (3,5-difluorophenacetyl)-L-alanyl-S-phenylglycine t-butyl ester
Db-cAMP	Dibutyryl-cyclic AMP
DE	Definitive endoderm
DM	Diabetes mellitus
ECCs	Endocrine cell clusters
ECM	Extracellular matrix
ECs	Endocrine cells
EGF	Epidermal growth factor
EPs	Endocrine progenitors
FAB	bFGF, Activin-A and BMP4
GIP	Glucose-dependent insulinotropic polypeptide
GLP1	Glucagon-like peptide-1
GLUT1/2	Glucose transporter ½
GSIS	Glucose stimulated insulin secretion
GSK-3 β	Glycogen synthase kinase-*3* beta
hAFSC	Human amniotic fluid-derived stem cells
hESCs	Human embryonic stem cells
HGF	Hepatocyte Growth Factor
hiPSCs	Induced pluripotent stem cells
hPSCs	Human pluripotent stem cells
ITS-X	Insulin, Transferrin, Selenium, Ethanolamine solution
KGF	Keratinocyte growth factor
NME2	Non-metastatic cells 2
pdECM	Pancreatic tissue derived ECM
PE	Pancreatic endoderm
PGT	Posterior gut tube
PI3K	Phosphoinositide 3-kinases
PKC	Protein kinase C
PPs	Pancreatic progenitors
RA	Retinoic acid
SHH	Sonic hedgehog
SIRT	Sirtulins
T3	T3 Triiodothyronine
TGF-β1	Transforming growth factor beta 1
TPB	(((2S,5S)-(E,E)-8-(5-(4-(trifluoromethyl)phenyl)-2,4 pentadienoylamino)benzolactam)
UCP2	Uncoupling protein 2
